# Prognostic factors influencing venous patency after thrombectomy in patients with May-Thurner syndrome

**DOI:** 10.1371/journal.pone.0336037

**Published:** 2025-11-25

**Authors:** Chang Hoon Oh, In Chul Nam, Doo Ri Kim, Jung Ho Won, Hyoung Nam Lee, Sung-Joon Park, Youngjong Cho

**Affiliations:** 1 Department of Radiology, Samsung Medical Center, Sungkyunkwan University School of Medicine, Seoul, Republic of Korea; 2 Department of Radiology, Jeju National University School of Medicine, Jeju Natuional University Hospital, Jeju, Republic of Korea; 3 Department of Radiology, Gyeongsang National University College of Medicine and Gyeongsang National University Hospital, Jinju, Republic of Korea; 4 Department of Radiology, Soonchunhyang University College of Medicine, Cheonan Hospital, Cheonan, Republic of Korea; 5 Department of Radiology, Korea University College of Medicine, Korea University Ansan Hospital, Ansan, Republic of Korea; 6 Department of Radiology, University of Ulsan College of Medicine, Gangneung Asan Hospital, Gangneung, Republic of Korea; Ataturk University Faculty of Medicine, TÜRKIYE

## Abstract

**Purpose:**

To evaluate the prognostic significance of venographic findings and procedural factors for long-term venous patency after thrombectomy in patients with May-Thurner syndrome (MTS)-associated deep vein thrombosis (DVT).

**Materials and Methods:**

This retrospective cohort study included 75 patients with iliofemoral DVT secondary to MTS, who underwent thrombectomy between January 2011 and April 2023. Key venographic findings—venous stenosis (≥50%), venous spur, and persistent collaterals—along with stent placement and diameter were analyzed. The primary outcome was 24-month venous patency, assessed using univariate tests, Kaplan–Meier survival analysis, and multivariate Cox proportional hazards modeling.

**Results:**

At 24 months, 58 patients (77.3%) maintained venous patency, whereas 17 (22.7%) experienced reocclusion. Univariate and Kaplan–Meier analyses showed that stenosis ≥50%, venous spur, persistent collaterals, and absence of stent placement were significantly associated with reduced patency (all *p* < 0.05). However, in the multivariate Cox model, only venous stenosis ≥50% remained a statistically significant independent predictor of reocclusion (hazard ratio [HR]=5.04; 95% Confidence Interval: 1.28–19.82; *p* = 0.021). Stent placement (HR = 1.78; *p* = 0.337) and diameter (*p* = 0.349) were not independently associated with patency.

**Conclusion:**

Residual venous stenosis ≥50% following thrombectomy is an independent predictor of reduced long-term patency in patients with MTS. While stent placement was associated with better outcomes in the univariate analysis, it did not independently predict patency after adjustment, likely due to treatment-related confounding factors. Effective anatomical resolution of stenosis may be more critical than stent deployment. Prospective studies are warranted to clarify the prognostic impact of venographic findings and interventional strategies.

## Introduction

May-Thurner syndrome (MTS), also known as iliac vein compression syndrome, is a vascular condition characterized by extrinsic compression of the left common iliac vein (LCIV) by the overlying right common iliac artery (RCIA) against the lumbar spine [1,2]. This chronic compression can lead to intimal hypertrophy, venous spur formation, and luminal narrowing, predisposing the affected individuals to iliofemoral deep vein thrombosis (DVT), especially in the left lower extremity [3]. Several anatomical variants have been reported, including compression of the LCIV by the left common iliac artery and right-sided variants such as right common iliac vein compression by the RCIA or right internal iliac artery [4,5]. Although some patients remain asymptomatic due to compensatory collateral flow, others may present with acute symptoms requiring prompt intervention to prevent post-thrombotic complications [6,7].

Over the past decade, endovascular approaches such as catheter-directed thrombolysis and pharmacomechanical thrombectomy have become the mainstay of treatment for iliofemoral DVT associated with MTS [8–13]. In cases of significant residual stenosis after thrombus removal, stent placement is often performed to restore venous patency and prevent re-thrombosis. However, the decision to place a stent is frequently based on intra-procedural venographic findings and operator preference, resulting in variability in clinical practice. Moreover, the prognostic significance of specific venographic features, such as luminal irregularity, persistent collaterals, and the degree of stenosis, has not been fully established. Although previous studies [14,15] have demonstrated favorable short- and long-term outcomes with iliac vein stenting in MTS with acute DVT, the predictors of sustained venous patency remain incompletely understood, especially in patients undergoing endovascular treatment.

Therefore, this study aimed to evaluate the prognostic impact of various venographic features, including venous stenosis, luminal irregularity, and persistent collaterals, on long-term venous patency after thrombectomy in patients with MTS. We also assessed whether stent placement and size were independently associated with primary patency.

## Materials and methods

The Institutional Review Board of our hospital approved this study (JEJUNUH 2025-05-012), and the requirement for informed consent was waived owing to the retrospective nature of the study.

### Study population

**Fig 1:**
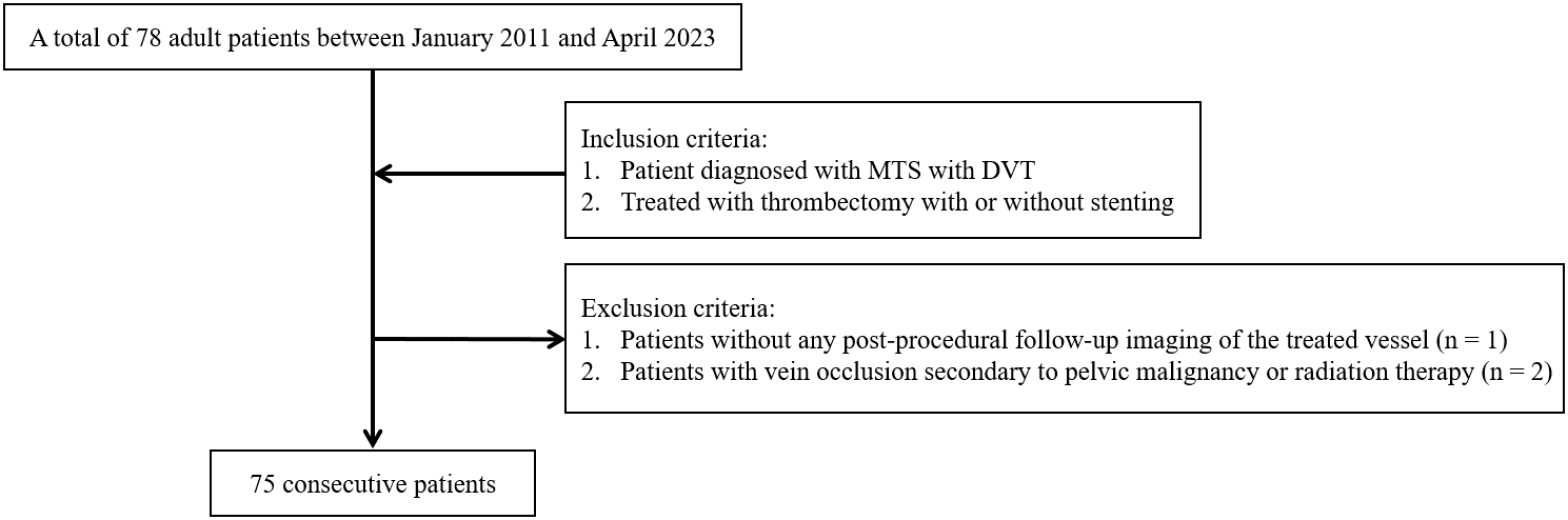
Case accrual process.

This retrospective cohort study was conducted at a single tertiary medical center. Clinical data were accessed for research purposes between 30/05/2025 and 30/06/2025. From January 2011 to April 2023, we identified 78 consecutive adult patients (aged ≥18 years) diagnosed with iliofemoral DVT secondary to MTS who underwent thrombectomy. Patients without follow-up imaging of the treated veins or those with malignancy-associated pelvic vein compression (n = 2) were excluded. Ultimately, 75 patients were included in the final analysis. All patients underwent initial contrast-enhanced lower extremity computed tomography (CT) venography to confirm the diagnosis of MTS and evaluate the extent of the thrombus. Various endovascular treatments, including catheter-directed thrombolysis and pharmacomechanical thrombectomy, were performed as primary interventions. After initial treatment, the degree of residual stenosis in the LCIV was assessed using conventional venography. Stent placement was performed selectively based on venographic findings and operator discretion. The case accrual process is illustrated in Fig 1.

### Follow-up

Anticoagulation therapy was initially managed with low-molecular-weight heparin, which was subsequently switched to daily oral warfarin for a minimum duration of six months. The goal was to maintain the international normalized ratio within the therapeutic range of 2–3 for the majority of the patients. All patients were scheduled for follow-up at 1, 6, and 12 months, and then annually as feasible. During the initial one-month follow-up, a physical examination and imaging diagnosis, such as duplex ultrasound of the ipsilateral iliofemoral venous system or contrast-enhanced CT scans, were conducted. In case of clinical failure during the initial one-month follow-up, which meant that the patient’s symptoms did not improve and recurrent DVT was detected on imaging modalities, the clinician decided on treatment according to their priority, either re-intervention or continued anticoagulation therapy alone.

### Intervention techniques

All procedures were performed by one of four interventional radiologists with 5, 7, 12, or 21 years of experience in venous intervention. The procedures were performed using an angiography suite (AlluraClarity FD20, Philips Healthcare, Best, Netherlands; Artis Zee Ceiling, Siemens Healthcare, Erlangen, Germany).

First, local anesthesia (10–15 mL of 2% lidocaine) was injected using a 22G needle along the entrance point around the left popliteal vein under US guidance and analgesia in the prone position. Venous access was achieved through the ipsilateral popliteal veins. Initially, a 14Fr sheath was inserted, followed by passage of a 0.035’‘ hydrophilic wire into the inferior vena cava using a 5Fr catheter. Subsequently, a mixture of iodine contrast and normal saline solution was carefully injected while gradually withdrawing the 5Fr catheter to evaluate the extent and burden of the thrombus in the ileofemoral vein and to assess the location and severity of the iliac vein obstruction. In all enrolled patients, mechanical thrombectomy was performed as the primary treatment; no patient underwent thrombolysis alone. After removal of most thrombotic material using manual aspiration or pharmacomechanical devices, balloon angioplasty was performed on the stenotic segment, followed by completion venography to assess flow restoration and residual obstruction. The decision to perform stent placement was based on venographic findings after angioplasty—particularly in cases showing persistent occlusion, inadequate flow restoration, or significant residual stenosis—and was ultimately left to the discretion and preference of the operating interventional radiologist at the time of the procedure. For thrombectomy, the hub of an 11Fr braided sheath was trimmed to enable connection to a syringe, after which multiple aspiration thrombectomies were performed to remove the majority of the thrombus, or an Angiojet (Boston Scientific, Marlborough, Mass, USA) catheter was employed for single-session pharmacomechanical thrombolysis. Subsequently, an 8–14 mm diameter balloon catheter was exchanged, and angioplasty was performed by inflating for 2–3 minutes at the usual 4–10 ATM. For stenting, self-expandable metal stents were used to cover the area of maximal compression, and stents were deployed with 2–3 mm of the cephalad end of the stent placed into the inferior vena cava to cover all disease segments. The stent diameter was based on relevant venography measurements; usually, a stent 10–18 mm in diameter was used, and undersizing was avoided.

### Data collection

**Fig 2:**
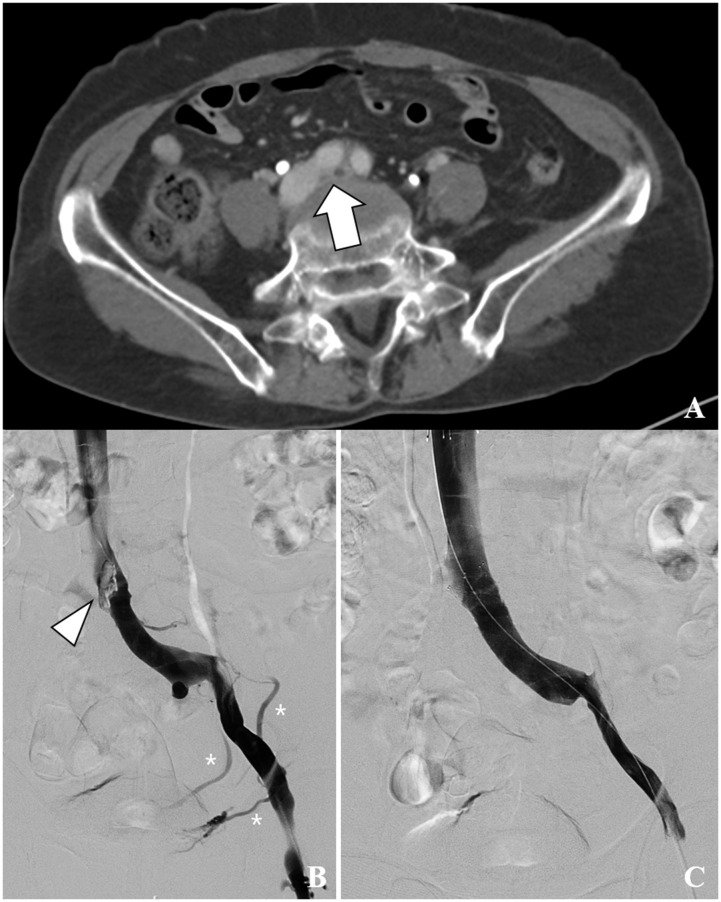
Endovascular treatment of May-Thurner syndrome-associated iliofemoral deep vein thrombosis in a 76-year-old woman. **A.** Contrast-enhanced computed tomography venography showing compression of the left common iliac vein (LCIV) by the overlying right common iliac artery and L4 vertebral body (arrow), consistent with May-Thurner syndrome. **B.** Conventional venography reveals a prominent venous spur and > 50% luminal narrowing at the LCIV (arrowhead), along with collateral venous flow (asterisk). **C.** Post-stenting venogram after the deployment of a 14-mm self-expandable stent demonstrating complete resolution of venous stenosis, spur formation, and collateral flow.

Data on age and sex were also collected. The intervals between symptom onset and treatment were also assessed. Venographic and procedural data were acquired to evaluate specific venous abnormalities associated with MTS. The following five binary variables were assessed: a) venous stenosis ≥ 50%, venous stenosis was defined as a ≥ 50% reduction in the diameter of the LCIV compared to the adjacent non-stenotic segment; b) venous spur (luminal irregularity), the presence of fibrotic spurs or nodular protrusions within the venous lumen on venography; c) persistent collaterals, defined as the continued visualization of collateral veins, indicating persistent venous outflow obstruction; d) stent placement during the procedure; e) stent diameter, which was dichotomized based on a threshold of 13 mm derived from receiver operating characteristic (ROC) curve analysis. Fig 2 demonstrates representative venographic findings demonstrating characteristic features of MTS.

### Statistical analysis

Categorical variables were compared using the chi-square test or Fisher’s exact test, and continuous variables were analyzed using the Student’s t-test. Categorical variables are expressed as frequencies and percentages, while continuous variables are expressed as means ± standard deviation. For each venographic finding, a 2 × 2 contingency table was constructed to evaluate the association between the presence of each finding and 24-month patency status (patent vs. occluded). Chi-square or Fisher’s exact test was used to calculate *p*-values for each comparison, with Fisher’s exact test used when the expected cell counts were < 5. To determine the optimal cutoff value for stent diameter as a prognostic factor for 24-month patency, ROC curve analysis was performed. The area under the curve was calculated to assess the discriminative power of stent size for predicting patency status. The optimal cutoff value was determined using Youden’s index (sensitivity + specificity – 1), identifying the stent diameter that maximized both sensitivity and specificity. Based on the identified cutoff, stent diameter was categorized as a binary variable (<cutoff mm vs. ≥ cutoff mm). Subsequent Kaplan-Meier and Cox proportional hazards analyses were performed using the binary stent diameter variable. Long-term patency was assessed using Kaplan-Meier survival analysis. Survival curves were generated based on statistically significant venographic findings and binary stent diameter variables identified through ROC analysis. The log-rank test was used to compare the survival curves, with the significance level set at *p* < 0.05. A Cox proportional hazards regression model was constructed to identify the independent prognostic factors associated with venous patency. Variables with *p* < 0.05 in univariate analysis and clinically significant factors were included in the multivariate model. Stepwise backward elimination was used to refine the model, and hazard ratios (HRs) with 95% confidence intervals (CIs) were calculated for each variable. Prior to inclusion in the Cox regression model, potential confounders were assessed for multicollinearity. The variance inflation factor was calculated to evaluate collinearity, and variables with variance inflation factor > 5 were excluded to prevent multicollinearity. All statistical tests were two-sided, and a *p*-value <0.05 was considered statistically significant. All statistical analyses were performed using IBM SPSS Statistics for Windows version 29 (IBM Corp., Armonk, N.Y., USA).

## Results

Finally, we included 75 patients diagnosed with symptomatic MTS and DVT who were treated with endovascular techniques. The study patients consisted of 18 (27.7%) men and 47 (72.3%) women (mean age, 65.4 ± 18.8 years). Table 1 summarizes the patient demographics.

**Table 1:** Baseline demographic characteristics of patients with symptomatic May-Thurner syndrome.

	Total (n = 75)
Mean age (years)	66.3 ± 17.9
Sex, n (%)	
Male	21 (32.3)
Female	54 (83.1)
Symptom onset to treatment (days)	6.5 ± 5.9
Symptom onset < 14 days	69 (92)
Symptom onset ≥ 14 days	6 (8)
Initial treatment modality	
Thrombectomy with stenting	45 (60)
Thrombectomy without stenting	30 (40)
Stent diameter (mm)	13.3 ± 1.7

Associations between venographic and procedural factors and the 24-month primary patency were evaluated using 2 × 2 contingency tables (Table 2). Among the 75 patients, 58 (77.3%) maintained venous patency at 24 months, whereas 17 (22.7%) developed reocclusion. Venous stenosis ≥50% was present in 20 patients, of whom 13 (65.0%) experienced occlusion. In contrast, among the 55 patients without significant stenosis, only 4 (7.3%) developed occlusion. This difference was statistically significant (*p* < 0.001). Venous spurs are also strongly associated with re-occlusion. Among the 32 patients with a spur, 15 (46.9%) experienced occlusion, compared to only 2 (4.7%) of the 43 patients without a spur (*p* < 0.001). Persistent collaterals were found in 5 patients, 4 of whom (80.0%) had occlusion. In contrast, among 70 patients without collateral flow, only 13 (18.6%) developed occlusion (*p* = 0.002). Stent placement was more common in patients with occlusions. Of the 30 patients without stent placement, 11 (36.7%) experienced occlusion, whereas among the 45 who received a stent, only 6 (13.3%) had occlusion (*p* = 0.018). Lastly, the stent diameter did not show a significant association with 24-month patency. Among the 22 patients with stents smaller than 12 mm in diameter, 4 (18.2%) experienced occlusion, compared to 2 (8.7%) of the 23 patients with larger stents (*p* = 0.349). Table 2 summarizes the results of the univariate analysis.

**Table 2:** Univariate analysis of venographic and procedural factors associated with 24-month primary patency.

		Patent (*n* = 58)	Occluded (*n* = 17)	*p* value
Venous stenosis	< 50%	51	4	< 0.001
	≥ 50%	7	13	
Venous spur	Absent	41	2	< 0.001
	Present	17	15	
Persistent collaterals	Absent	57	13	0.002
	Present	1	4	
Stent placement	No	19	11	0.018
	Yes	39	6	
Stent diameter (only stent group, *n* = 45)	≤ 12 mm	18	4	0.349
	≥ 14 mm	21	2	

**Fig 3:**
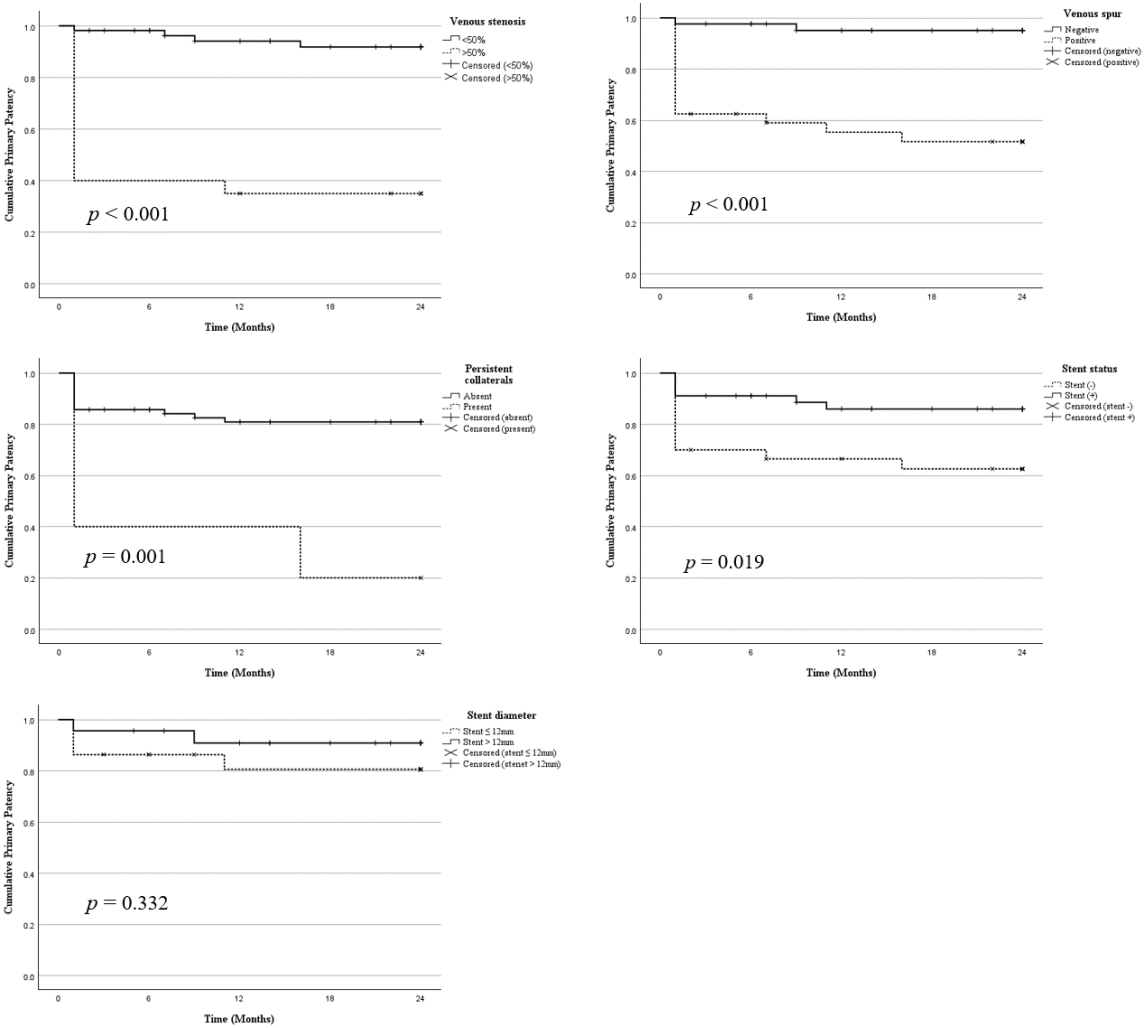
Kaplan–Meier curve showing 24-month cumulative primary patency stratified by venographic and procedural factors. Each graph shows the cumulative primary patency rates among patients with and without specific risk factors. Differences in the survival distribution were assessed using log-rank tests. **A.** Venous stenosis. Patients with stenosis ≥50% showed significantly lower patency compared to those with <50% stenosis (*p* < 0.001). **B.** Venous spur. Presence of luminal irregularity (spur) was associated with inferior patency outcomes (*p* < 0.001). **C.** Persistent Collateral Veins. Patients without collateral flow demonstrated significantly higher patency rates than those with persistent collateral flow (*p* = 0.001). **D.** Stent placement. Patients who underwent stent placement had a higher long-term patency than those who did not (*p* = 0.019). **E.** Stent diameter. Among patients with stent placement, no significant difference in patency was observed between stents ≤12 mm and >12 mm in diameter (*p* = 0.332).

Kaplan–Meier survival analysis was performed to assess cumulative primary patency rates according to key venographic and procedural factors. Patients without significant stenosis (stenosis <50%) demonstrated a significantly higher cumulative primary patency rate than those with stenosis ≥50% (Fig 3A). Similarly, the absence of a venous spur was associated with superior long-term patency, with the spur-negative group showing significantly better survival than the spur-positive group (Fig 3B). Persistent collateral veins are also associated with poor outcomes. Patients without collateral flow showed significantly higher patency rates than those with persistent collateral flow (Fig 3C). Regarding the procedural factors, stent placement was associated with improved cumulative patency. The stent group exhibited a significantly higher patency over 24 months than the non-stent group (Fig 3D). However, within the stent group, Kaplan–Meier analysis stratified by stent diameter (≤12 mm vs. > 12 mm) showed no statistically significant differences in patency outcomes (Fig 3E).

To identify independent prognostic factors associated with 24-month primary venous patency, a multivariate Cox proportional hazards regression analysis was performed, including stenosis ≥50%, spur, persistent collaterals, and stent placement as covariates (Table 3).

**Table 3:** Multivariate cox proportional hazards regression analysis for a 24-month primary patency.

	Hazard ratio	95% CI	*p* value
Venous stenosis	5.04	1.28–19.82	0.021
Venous spur	5.33	0.85–33.49	0.074
Persistent collaterals	1.90	0.55–6.56	0.310
Stent placement	1.78	0.55–5.80	0.337

CI, confidence interval.

Among these variables, stenosis ≥50% emerged as the only statistically significant independent predictor of venous occlusion (HR = 5.04; 95% CI: 1.28–19.82; *p* = 0.021). This indicates that patients with significant residual stenosis had an over fivefold increased risk of reocclusion compared with those without. The presence of venous spur showed a borderline association with occlusion risk (HR = 5.33; CI: 0.85–33.49; *p* = 0.074), suggesting a potential trend but not reaching statistical significance. Persistent collaterals (HR = 1.90, 95% CI: 0.55–6.56; *p* = 0.310) and stent placement (HR = 1.78, 95% CI: 0.55–5.80; *p* = 0.337) were not found to be independently associated with long-term patency in the adjusted model.

## Discussion

Cross-sectional imaging in MTS often reveals engorged collateral vessels bypassing the compressed segment, which is a characteristic feature suggestive of MTS [7]. Although variants in compressive anatomy have been described, including right-sided or bilateral forms, the classical pattern involves LCIV impingement by the RCIA [4,5]. In recent years, therapeutic strategies for thrombotic MTS have increasingly favored endovascular approaches, such as catheter-directed thrombolysis and pharmacomechanical thrombectomy with or without stent placement, owing to their minimally invasive nature and effectiveness in restoring venous patency [8–13,16–20]. This paradigm shift has also been driven by the need to mitigate long-term complications such as post-thrombotic syndrome, which can significantly impair quality of life in affected patients [21,22]. This retrospective cohort study investigated the prognostic significance of specific venographic and procedural findings observed at the time of venography completion after thrombectomy in patients with MTS-associated iliofemoral DVT. Particular emphasis was placed on evaluating whether anatomical features, such as residual venous stenosis, luminal irregularity, or persistent collateral flow, could predict long-term venous patency. In addition, the impact of stent placement and diameter on the outcomes was assessed to clarify whether procedural factors independently contributed to sustained vein patency over a 24-month follow-up period.

In this study, among the assessed variables, only the presence of ≥50% venous stenosis on completion venography emerged as an independent predictor of reduced long-term patency in the multivariate analysis, whereas other findings, such as venous spur, persistent collaterals, and stent placement, were not statistically significant. The observation that venous stenosis ≥50% was significantly associated with reduced patency aligns with existing literature underscoring the importance of anatomical obstruction in venous flow impairment and thrombosis recurrence [23]. Even after a successful thrombectomy, residual stenosis may promote turbulent flow, venous hypertension, and subsequent re-thrombosis. Our findings emphasize that the persistence of significant stenosis, regardless of stent placement, remains a key prognostic factor and warrants meticulous intraprocedural assessment.

Interestingly, stent placement itself was not independently associated with patency in our Cox regression model despite demonstrating significance in the univariate and Kaplan–Meier analyses. This discrepancy is likely attributable to the treatment-induced modification of the disease process. In our study, stent placement was not randomized but was instead determined by operator discretion based on venographic findings. It is plausible that patients who received stents had more severe anatomical abnormalities, such as high-grade stenosis or persistent collaterals, which were corrected through the intervention. Consequently, favorable long-term outcomes may reflect successful anatomical correction rather than stenting. Similar observations have been reported previously [23,24]. Kim et al. [23] showed that in-stent thrombosis, rather than initial anatomical severity, was a major predictor of recurrent DVT, highlighting the prognostic shift introduced by treatment. The pathophysiological process leading to thrombus formation after venous stent placement is multifactorial, involving hemodynamic disturbance, endothelial injury, and patient-related factors. Following stent deployment, areas of turbulent or stagnant flow can develop at the stent edges or within incompletely expanded segments, promoting local endothelial injury and platelet activation, which initiate thrombus formation [25]. Inadequate stent expansion or incomplete coverage of the diseased segment may result in residual stenosis and venous stasis, further increasing thrombogenic potential [26]. In addition, the metallic surface of the stent can provoke local inflammation until complete endothelialization occurs, contributing to a prothrombotic microenvironment [27]. Systemic conditions, such as hypercoagulability, immobility, dehydration, or suboptimal anticoagulation, can exacerbate this process [28]. These mechanisms together explain why in-stent thrombosis remains an important clinical concern despite technically successful stent placement. This phenomenon illustrates a key challenge in retrospective analyses of interventional outcomes, where therapeutic decisions alter the underlying pathology, thereby limiting the ability to assess the natural history and pure prognostic value of the anatomical findings. Similarly, Raju and Neglén [24] emphasized that the resolution of anatomical obstruction, rather than the act of stenting itself, was a key factor in improving venous hemodynamics and clinical outcomes. This treatment-related confounding factor may partly explain why stenting, a definitive corrective measure, was not an independent prognostic factor in multivariate analysis. In other words, once stenosis was successfully resolved by stenting, the prognostic weight of that anatomical feature was eliminated, resulting in equalized outcomes between stented and non-stented patients. These findings support the notion that anatomical resolution of stenosis is more important than the procedural act of stenting. Although stent placement was not identified as an independent prognostic factor in the multivariate analysis, its association with improved patency in the univariate and Kaplan–Meier analyses suggested a potential benefit in selected patients. This observation likely reflects effective anatomical correction in patients with severe venous stenosis. Therefore, in clinical practice, timely stent placement based on venographic findings may play a critical role in optimizing long-term outcomes.

The presence of venous spurs and persistent collaterals was associated with patency loss in univariate and Kaplan–Meier analyses; however, these findings were not significant in multivariate modeling. This suggests that these features, while indicative of chronic venous compression or altered flow patterns, act more as surrogate markers of hemodynamic compromise than as independent pathologic drivers. Moreover, the high collinearity between spur formation and underlying stenosis may have attenuated their independent contribution to prognosis after statistical adjustment.

Regarding the stent diameter, we attempted to determine an optimal binary cutoff using ROC analysis, which yielded a threshold of 13 mm. However, the dichotomized variable did not reach statistical significance in either univariate or multivariate analyses, indicating that within the diameter ranges used in our cohort (10–18 mm), stent size may not be a major determinant of long-term patency. These findings may reflect the predominance of other anatomical and technical factors in the diameter selection to ensure successful venous decompression. This interpretation is consistent with the findings of Bozkaya, et al. [29], who reported favorable outcomes following endovascular treatment for MTS using various stent sizes, but did not observe or analyze outcome differences by stent diameter, suggesting that stent deployment itself, rather than its exact size, may be a more critical factor in clinical success.

This study had several limitations. First, its retrospective nature and single-center design may have introduced a selection bias and limited generalizability. Second, the decision to place a stent was not standardized, raising the possibility of unmeasured confounding factors. Third, stenting itself likely alters the natural course of the underlying anatomical abnormalities, particularly in patients with high-grade stenosis or persistent collaterals. As a result, it becomes difficult to disentangle whether favorable outcomes were driven by the anatomical correction achieved through stenting or would have occurred regardless. This phenomenon represents an inherent limitation of retrospective designs, in which treatment modifies the variable under investigation, thereby obscuring its independent prognostic value. To better delineate the causal impact of stent placement versus anatomical severity, a prospective cohort study with clearly defined criteria for intervention and stratification based on preintervention findings would be more informative. Finally, the sample size, although relatively large for a single-center MTS cohort, may limit the ability to detect weaker prognostic signals.

In conclusion, our study demonstrates that residual (≥50%) venous stenosis on completion venography is a significant independent predictor of reduced long-term patency following endovascular treatment for MTS-associated DVT. Although stent placement was not an independent prognostic factor in multivariate analysis, it may contribute to favorable outcomes by alleviating anatomical obstruction. These findings suggest that procedural success in MTS should be judged not solely by stent deployment, but also by the effective resolution of venous stenosis. Future prospective studies with standardized imaging protocols and postprocedural evaluations are needed to clarify the interplay between anatomical findings, interventional decisions, and long-term outcomes.
